# Differences in genomic abnormalities among African individuals with monoclonal gammopathies using calculated ancestry

**DOI:** 10.1038/s41408-018-0132-1

**Published:** 2018-10-10

**Authors:** Linda B. Baughn, Kathryn Pearce, Dirk Larson, Mei-Yin Polley, Eran Elhaik, Michael Baird, Colin Colby, Joanne Benson, Zhuo Li, Yan Asmann, Terry Therneau, James R. Cerhan, Celine M. Vachon, A. Keith Stewart, P. Leif Bergsagel, Angela Dispenzieri, Shaji Kumar, S. Vincent Rajkumar

**Affiliations:** 1 Division of Laboratory Genetics, Department of Laboratory Medicine and Pathology, Mayo Clinic, Rochester, MN USA; 2 Division of Biomedical Statistics and Informatics, Department of Health Sciences Research, Mayo Clinic, Rochester, MN USA; 3 Department of Animal and Plant Sciences, University of Sheffield, Sheffield, UK; 4 DNA Diagnostics Center, Fairfield, OH USA; 5 Department of Health Sciences Research, Mayo Clinic, Rochester, MN USA; 6 Division of Hematology, Department of Internal Medicine, Mayo Clinic, Scottsdale, AZ USA; 7 Division of Hematology, Department of Internal Medicine, Mayo Clinic, Rochester, MN USA

## Abstract

Multiple myeloma (MM) is two- to three-fold more common in African Americans (AAs) compared to European Americans (EAs). This striking disparity, one of the highest of any cancer, may be due to underlying genetic predisposition between these groups. There are multiple unique cytogenetic subtypes of MM, and it is likely that the disparity is associated with only certain subtypes. Previous efforts to understand this disparity have relied on self-reported race rather than genetic ancestry, which may result in bias. To mitigate these difficulties, we studied 881 patients with monoclonal gammopathies who had undergone uniform testing to identify primary cytogenetic abnormalities. DNA from bone marrow samples was genotyped on the Precision Medicine Research Array and biogeographical ancestry was quantitatively assessed using the Geographic Population Structure Origins tool. The probability of having one of three specific subtypes, namely t(11;14), t(14;16), or t(14;20) was significantly higher in the 120 individuals with highest African ancestry (≥80%) compared with the 235 individuals with lowest African ancestry (<0.1%) (51% vs. 33%, respectively, *p* value = 0.008). Using quantitatively measured African ancestry, we demonstrate a major proportion of the racial disparity in MM is driven by disparity in the occurrence of the t(11;14), t(14;16), and t(14;20) types of MM.

## Introduction

Monoclonal gammopathies, such as multiple myeloma (MM), represent a collection of plasma cell (PC) neoplasms comprised of mostly incurable hematopoietic malignancies with an increasing incidence (~6 cases per 100,000 individuals during 2008–2012) in the United States^1,2^. MM is the most common hematologic malignancy in African Americans (AAs). AAs have a 2–3-fold higher prevalence of monoclonal gammopathy of undetermined significance (MGUS) and a similarly higher incidence of MM, along with a ~4-year younger age of onset compared to European Americans (EAs)^3^. The increased incidence of MM among AAs has been attributed to the increased prevalence of MGUS, with a similar risk of MGUS to MM progression between EAs and AAs^3^. Increased MGUS prevalence cannot be fully explained by environmental differences between AAs and EAs in the US, since West African Ghanaian men also display increased prevalence of MGUS^4^. The combined observations that MGUS/MM clusters in families observed by a 2–4-fold increased risk of first-degree relatives of MM^5–7^, the higher incidence of MGUS among AAs and Western Africans and the earlier age of onset of MM in AAs suggest an ancestral-associated genetic predisposition to developing MM^8^. Further, when access to care is equal, AAs have better overall survival compared to EAs, suggesting that AAs may have a genetic predisposition that renders them better responders to treatment or have more indolent subtypes of MM^9^.

MM, although considered a single disease, can be divided into different cytogenetically defined subtypes with differences in disease outcome. Cytogenetic subtypes include hyperdiploidy (characterized by gains of odd-numbered chromosomes), and translocations involving the immunoglobulin heavy chain (*IgH*) gene on chromosome 14 with partner chromosomes resulting most commonly in t(11;14), t(4;14), t(14;16) and more rarely *IgH* translocations involving t(6;14), and t(14;20). The primary cytogenetic abnormalities most associated with standard risk includes hyperdiploidy; t(11;14) or t(6;14) and high-risk abnormalities are defined as t(4;14), t(14;16) and t(14;20)^10,11^. Secondary cytogenetic findings, including gain of chromosome 1q, deletion of 17p, including the *TP53* gene and rearrangements involving the *MYC* locus can also influence disease outcome^12^. Previous studies reported that AAs exhibit a lower frequency of *IgH* translocations, including reduced t(11;14) and t(4;14) in some studies, and no significant differences in hyperdiploidy were observed^13,14^. Most of these studies, however, relied on self-reported race, which is known to be a highly biased measure rather than genetic ancestry ^15–17^. Leveraging ancestry informative single-nucleotide polymorphisms (SNPs) allows quantifying one’s genetic ancestry in an admixture framework. We hypothesize that quantifying genetic ancestry is a necessary component to fully understand the genetic mechanisms of racial disparities of monoclonal gammopathies. In this study, we utilized genotyping data to calculate individual ancestry and examined whether primary and secondary cytogenetic abnormalities differed by high and low African ancestry.

## Materials and Methods

### Sample eligibility

Samples for this study were obtained from the Mayo Clinic Genomics Laboratory after obtaining Institutional Review Board approval. A retrospective cohort of 1000 specimens were identified from patients who had an abnormal plasma cell proliferative disorder fluorescence in situ hybridization (FISH) result and a concurrent conventional G-banded chromosome evaluation as part of routine clinical testing. The abnormal plasma cell FISH result along with patient age at the time of clinical cytogenetic testing, gender and self-reported race (if available) were also recorded.

### Fluorescence in situ hybridization (FISH) analysis

Plasma cell proliferative disorder FISH of immunoglobulin (cIg)-stained positive PCs studies were performed as part of routine clinical testing using the following probes: *RB1*/LAMP1 (Abbott Molecular, Des Plains, IL, USA) for monosomy 13 or 13q deletion, *TP53*/D17Z1 (Abbott Molecular) for *TP53* deletion or monosomy 17, D3Z1/D7Z1/D9Z1/D15Z4 (Abbott Molecular) for trisomy 3, 7, 9 or 15, *TP73*/1q22 (custom probe) for 1q gain, *MYC* (Abbott Molecular) for 8q24.1 rearrangement, *IgH* (custom probe) for 14q32 rearrangements and probes targeting individual *IGH* rearrangement detecting t(11;14)(q13;q32) *CCND1/IgH* (Abbott Molecular), t(4;14)(p16.3;q32) *FGFR3*/*IgH* (Abbott Molecular), t(6;14)(p21;q32) *CCND3*/*IgH* (custom probe), t(14;16)(q32;q23) *IgH*/*MAF* (Abbott Molecular), and t(14;20)(q32;q12) *IgH*/*MAFB* (custom probe). Deletion or monosomy of chromosomes 13 and 17 and copy number gain of 1q were detected using enumeration strategy probes. Centromere probes were used to detect chromosomal aneusomy of chromosomes 3, 7, 9, and 15. Translocations involving *IgH* with *FGFR3*, *CCND1*, *CCND3, MAF*, and *MAFB* were detected using dual-color, dual-fusion (D-FISH) strategy probes and rearrangements of *IgH* and *MYC* were detected using a break-apart strategy (BAP) probe. Plasma cells were identified using immunoglobulin staining techniques using antibodies targeting cytoplasmic immunoglobulin kappa and lambda. For each probe set, 50 plasma cells (if possible) were scored and the result for each probe was reported.

### Chromosome analysis

A conventional G-banded chromosome evaluation was performed as part of routine clinical testing. First, a cell count is performed on the specimen to establish a plating volume and based on the cell count, a corresponding volume of bone marrow is added to 2 culture flasks containing culture medium and incubated for 24 to 48 h at 37 degrees C. In the harvest process, the cells are exposed to colcemid and hypotonic solution, and are fixed with glacial acid and methanol. Metaphase cells are dropped onto microscope slides and are stained by G-banding and twenty metaphases are usually examined. Minimal evidence for the presence of an abnormal clone is defined as 2 or more metaphases with the same structural abnormality or chromosome gain (trisomy), or 3 or more metaphases lacking the same chromosome. All cells analyzed are captured using a computerized imaging system, and 1 or more karyograms from each clone are prepared to document the type of abnormality and to permit systematic interpretation of the anomalies. For the purpose of this study, loss of the Y chromosome and presumed constitutional abnormalities such as inv(2)(p11.2q13) were interpreted as a normal result. A portion of the cell culture pellet is fixed in methanol/acetic acid for storage.

### DNA extraction and PMRA genotyping

DNA was isolated from fixed cell pellets from residual chromosome studies that yielded normal results using the DNeasy Blood and Tissue Kit (Qiagen, Germantown, MD, USA) following the manufacturer’s recommended protocol. DNA was quantitated using a Qubit Fluorometric Quantitation Instrument (Thermo Fisher Scientific, Waltham, MA, USA) and 100 ng of DNA (5 ng/μL) was used for genotyping on a 96-well Axiom array manufactured by Affymetrix (Thermo Fisher Scientific), the Precision Medicine Research Array (PMRA) (https://www.thermofisher.com/order/catalog/product/902981) comprised of ~730,000 autosomal single-nucleotide polymorphisms (SNPs), following the manufacturer’s recommended protocol. A negative and two positive controls (Coriell samples) were included on each run. Data were analyzed by the Affymetrix Axiom Analysis Software Suite to determine genotypes with a required call rate threshold of at least 99%. The data from autosomal markers were analyzed by the GPS Origins Software (https://homedna.com/ancestry/gps-origins) to generate the ethnic breakdown of each sample.

### Biogeographical inference

Biogeographical analyses were carried out using the commercial Geographic Population Structure Origins (GPSO) tool provided by the DNA Diagnostics Center. GPSO works similarly to the GPS tool^18–20^, which calculates the ancestry of an individual in relation to nine putative ancestral populations representing geographic regions (e.g., South Africa) and outputs admixture proportions corresponding to those ancestries^19^. GPSO expands the original GPS model by inferring ancestry using 36 admixture proportions (Table 1) and was used to elucidate the African and non-African ancestries of each sample from the PMRA genotype data. The ancestry of the 881 samples was calculated by applying the GPSO to the SNP data genotyped on the Precision Medical Research Array (PMRA). GPSO provided an ancestral breakdown of 36 admixture components for each individual representing different geographic regions (Table 1). African ancestry was calculated by summing the ten ancestral African components (Table 1, populations 1–10) and European ancestry was similarly calculated using seven admixture components associated with Northern Europe and the Mediterranean (populations 28–34) (Table 1).

**Table 1:** Regional ancestries or admixture components employed by the GPSO algorithm

Regional ancestry		Description
*Africa*		
1	South African Bushmen	Localized to South Africa
2	African Pygmies	Associated with the Pygmy people
3	South western Africa	Peaks in Nigeria and declines in Senegal, Gambia, and Kenya
4	Hadza	Peaks in Tanzania
5	Madagascar	Peaks in Madagascar with residues in South Africa
6	Western Ethiopia	Peaks in Western Ethiopia and south Sudan
7	Northwestern Africa	Peaks in Algeria and declines in Morocco and Tunisia
8	Southern Ethiopia	South Ethiopia
9	South Africa	Peaks in Botswana, Namibia, Anglola, and with residues in South Africa
10	West Africa	Peaks in Senegal and Gambia and declines in Algeria and Morocco
*Native America*		
11	Central America	Peaks in Mexico and Central America with resides in Greenland, Peru, Siberia, and east Russia
12	Eastern Amazon	Associated with the Surui people in Brazil. Declines in Colombia
13	Pima County	Peaks in Central-North America and declines towards Greenland and Eskimos
14	Western Amazon	Peaks in endemic to the Karitiana people (Brazil) and declines in Colombia
15	Southeastern America	Peaks in Peru, Mexico, and North America and declines in Eastern Russia
*India*		
16	Northern India	Peaks in North India (Dharkars, Kanjars) and declines in Pakistan
17	Southeastern India	South eastern India with residues in Pakistan
18	Southwestern India	Peaks in Pulayar Indian with residues in Paniya, Savara, Bengali, Juang, Savara, Ho, Bonda Indian
*Southeast Asia*		
19	South China	Peaks in Taiwan and Malay and declines in Thailand, Vietnam, Cambodia, and South China
20	South Eastern Asia	Peaks in East Asia, Central-south China (Lahu, Naxi, Yi) and declines towards India
21	Central Southern China: Yunnan and Guangxi	Peaks in East Asia (East) and Chinese (She, Dai) with residues in Central south China (Han, Miao, Tujia)
22	North eastern Oceania	Peaks in Korea, Chinese (Han), Mynamar, Japan, and Vietnam and declines towards west China and India
*Northeast Asia*		
23	Japan	Peaks in Japan
24	Northeastern China	Peaks in East Asia and North East and declines in North east Russia and Siberia
25	North Mongolia	Peaks in north Mongolia and declines in Siberia
*Oceana*		
26	Bougainville	Peaks in Bougainville and declines in Australia
27	Papuan New Guinea	Peaks in Papua New Guinea and declines in Australia
*Northern Europe*		
28	Fennoscandia	Peaks in the Iceland and Norway and declines in Finland, England, and France
29	Orkney Islands	Peaks in the Orkney islands and declines in England, France, Germany, Belarus, and Poland
*Mediterranean*		
30	Arabia	Peaks in Saudi Arabia and Yemen and declines in Israel, Jordan, Iraq, and Egypt
31	Basque Country	Peaks in France and Spain Basque regions and declines in Spain, Sweden, France, and Germany
32	Sardinia	Peaks in Sardinia and declines in Italy, Greece, Albania, and The Balkans
33	Southern France	Peaks in south France and declines in France, England, Orkney islands, and Scandinavia
34	Eastern Mediterranean	Peaks in Israel with residues in Syria
*Siberia*		
35	Western Siberia	Peaks in Krasnoyarsk Krai and declines towards east Russia
36	East Russia	Peaks in South Siberia (Russians: Tuvinian) and declines in North Mongolia

### Statistical analysis and calculation of odds ratios

The analysis focused on examining the associations between the genetic abnormalities and African ancestry. The latter was examined as both a continuous variable (percentage of African genetics) and a categorical variable (primarily African descent, primarily European descent, and other). First, the association of the various genetic abnormalities and African ancestry (continuous) was examined using logistic regression in a generalized additive model; odds ratio estimates (and 95% confidence intervals) associated with 10% increase in African genetics were estimated. Smoothing spline was used to visualize the relationship between percentage of African genetics and probability of genetic abnormalities. Patients were also divided into 3 ancestral categories: African descent = at least 80% African ancestry; European descent = less than 0.1% African ancestry and <30% Asian ancestry; Other = all other genetic backgrounds; and the association of these categories with demographic factors and genetic abnormalities were evaluated using chi-square tests. Where appropriate, *p values* were adjusted using the Benjamini-Hochberg procedure to control the false discovery rate. All analyses were performed using R version 3.4.2.

## Results

### Patient cohort

Of the 1000 samples eligible for this study, genotype and ancestry data were obtained from 881 independent samples. All 881 samples had an abnormal plasma cell FISH result and had either a normal (*N* = 851) or abnormal (*N* = 30) chromosome study. The median age for the entire cohort at the time of cytogenetic testing was 64 years (range 26–90 years) with the highest proportion of individuals (35.4%) in the 60–69 age category. There were 478 males (54.3%) and 403 females (45.7%) with no significant difference in the proportion of primary cytogenetic abnormalities observed between males and females (Table 2). From the 881 samples, self-reported race was available from 393 individuals and 161 self-reported as African (including African American, Black or Caribbean black) and 185 self-reported as non-Hispanic Caucasian. Of the remaining 47 individuals from the self-reported cohort, 35 individuals identified as Asian, nine as Caucasian with Hispanic ethnicity, two as Native American and one self-reported with unknown ancestry. Self-reported ancestry information was not available from the remaining 488 individuals.

**Table 2:** Demographics by ancestry and cytogenetic abnormalities by gender

	African descent (*N* = 120)	European descent (*N* = 235)	Other (*N* = 526)	Total (*N* = 881)	*p* value
Demographics by ancestry					
*Gender*					0.028
Female	68 (56.7%)	99 (42.1%)	236 (44.9%)	403 (45.7%)	
Male	52 (43.3%)	136 (57.9%)	290 (55.1%)	478 (54.3%)	
*Age group*					0.096
<40	5 (4.2%)	3 (1.3%)	13 (2.5%)	21 (2.4%)	
40–49	8 (6.7%)	13 (5.5%)	50 (9.5%)	71 (8.1%)	
50–59	32 (26.7%)	57 (24.3%)	138 (26.2%)	227 (25.8%)	
60–69	47 (39.2%)	79 (33.6%)	186 (35.4%)	312 (35.4%)	
70–79	19 (15.8%)	69 (29.4%)	107 (20.3%)	195 (22.1%)	
80+	9 (7.5%)	14 (6.0%)	32 (6.1%)	55 (6.2%)	

	Female (*N* = 403)	Male (*N* = 478)	Total (*N* = 881)	*p* value
Abnormality by gender				
*Abnormality*				
t(11;14), t(14;16), or t(14;20)	135 (33.5%)	183 (38.3%)	318 (36.1%)	0.509
t(4;14)	39 (9.7%)	33 (6.9%)	72 (8.2%)	0.509
t(6;14)	7 (1.7%)	7 (1.5%)	14 (1.6%)	0.848
Other IgH	38 (9.4%)	48 (10.0%)	86 (9.8%)	0.848
Trisomy no IgH	157 (39.0%)	180 (37.7%)	337 (38.3%)	0.848
All Other	27 (6.7%)	27 (5.6%)	54 (6.1%)	0.848

*P* values are based on the comparison of the indicated abnormality (versus otherwise) compared to individuals ≥80.0% African ancestry, individuals with <0.1% African (excluding Asian ancestry) and all others individuals (3-group test, top table) and also to gender (bottom table) and are adjusted to control the false discovery rate (FDR) using the method of Benjamini and Hochberg

### Characterization of genetic ancestry

**Fig. 1:**
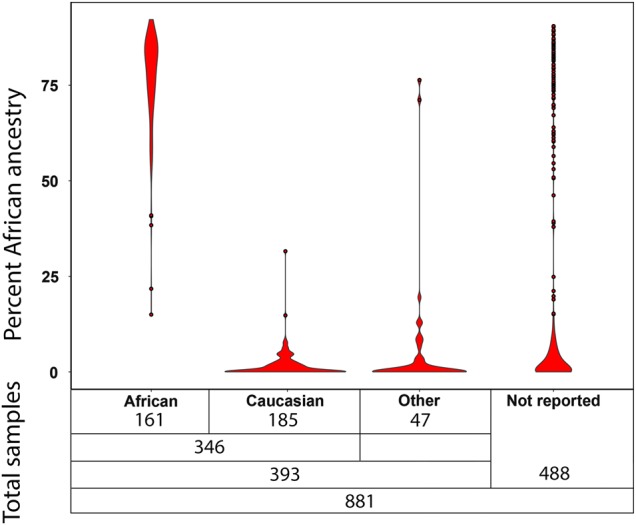
Percent African ancestry by self-reported race in cohort of 881 individuals. Distribution of the percent of African ancestry based on the sum of all 10 African regional ancestries within the 881 samples in this study by self-report race in 393 samples or non-reported race information in 488 samples

We first compared the calculated ancestry data to the self-reported ancestry information in the 393 individuals described above (Fig. 1). Of the 185 self-reported non-Hispanic Caucasian individuals, the median European ancestry was 68.2% (mean 67.9%, range 45.1–82.8%) [median Northern European 33.1% (mean 31.9%, range 15.8–44.5%)]. One self-reported Caucasian individual (omitted from the range calculation) had <0.1% European ancestry with 85.5% Asian ancestry. The median African admixture in the self-reported non-Hispanic Caucasian population was 0.30% (mean 1.6%, range 0–31.6%). Nearly all of those self-reporting as Caucasian (98.9%) had 8.6% or less African ancestry with the exception of two individuals with African ancestry of 14.8% and 31.6%. Of the 161 self-reported African individuals, the median African ancestry was 80% (mean 76.6%, range 15.0–92.2%). A self-reported African individual with the lowest African ancestry of 15.0% had 22.6% Native American and 36.2% European ancestry. The median European admixture in the self-reported African population was 5.8% (mean 8.8%, range 0–41.9%) [median Northern European 2.7% (mean 4.2, range 0–25.4%)]. While the range of African ancestry in individuals self-reporting as African was large, 98.8% of self-reported Africans had at least 30% African ancestry and 96.9% had at least 50% African ancestry.

Of the entire cohort of 881 individuals, the median African ancestry was 2.3% (mean 23.5%, range 0–92.2%), the median European ancestry was 64.7% (mean 47.6%, range 0–82.8%) and Northern European ancestry was 26.6% (mean 21.8%, range 0–44.5%) (Fig. 1). There were 268 individuals (30.4% of the entire cohort) with <0.1% African ancestry and 235 of these individuals also had <30% Asian ancestry representing our non-African and non-Asian cohort of Caucasian European individuals and 120 individuals (13.6%) had ≥80.0% African ancestry.

### Comparison of demographics and cytogenetic abnormalities using calculated ancestry

**Fig. 2:**
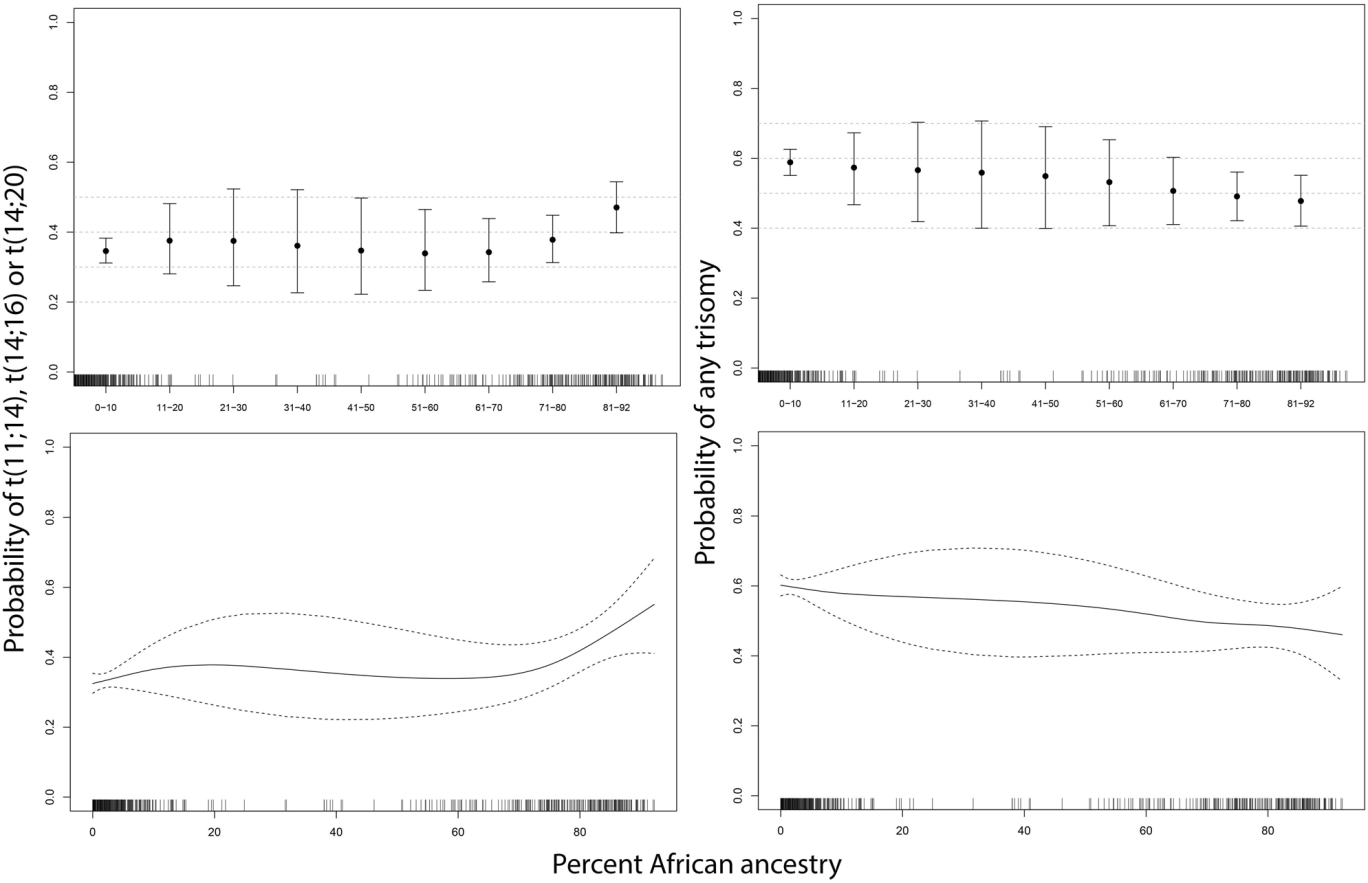
Probability of either t(11;14),t(14;16) or t(14;20) or any trisomy in relation to percent African ancestry. Smoothing spline was used to visualize the relationship between percentage of African genetics and probability of of t(11;14), t(14;16) or t(14;20) or of any trisomy

The prevalence of demographic variables and cytogenetic abnormalities was evaluated with respect to the percentage of African ancestry in the entire cohort. We first examined whether an increase in the percentage of African Ancestry altered the odds of any primary cytogenetic abnormality. The logistic regression model demonstrated that a 10% increase in the percentage of African ancestry was associated with a 6% increase in the odds of detecting either an t(11;14), t(14;16) or t(14;20) (odds ratio = 1.06, 95% CI: 1.02–1.11; *p* value = 0.05) (Table 3). Since we observed an increase in the prevalence of each of the individual t(11;14), t(14;16) and t(14;20) cytogenetic abnormalities with respect to African ancestry (Table 3), these three abnormalities were combined in downstream analysis. When we plotted the probability of observing these cytogenetic abnormalities with respect to the percentage of African ancestry (Fig. 2), we observed an increased probability of detecting either an t(11;14), t(14;16) and t(14;20) as well as reduced probability of observing an odd numbered trisomy (defined as having a gain of at least one of the following odd numbered chromosomes 3, 7, 9, 11, 15 and 17). The differences were most striking in the extreme populations, specifically among individuals with ≥80.0% African and individuals with <0.1% African ancestry (Fig. 2). On the basis of these results, we further evaluated the proportion of each cytogenetic abnormality within these most extreme cohorts with respect to African ancestry; individuals with ≥80.0% African and individuals with <0.1% African ancestry. A statistically significant higher prevalence of t(11;14), t(14;16) and t(14;20) (*p* value = 0.008) with a lower prevalence of trisomies (with or without IgH translocations) (*p* value = 0.066) was observed in the cohort with the greatest proportion of African ancestry (>80%) compared to the European cohort (≥0.1% African ancestry) (Table 4). In addition, the >80% African ancestry cohort also had statistically significant lower prevalence of monosomy 13/13q deletion (*p* value = 0.021) (Table 5) and a significantly higher prevalence in the proportion of females with monoclonal gammopathies compared to the European cohort (*p* value = 0.028) (Table 2). Similar to previous studies^9,21^, we identify an approximate two-fold reduction in the number of individuals that are ≥80.0% African compared to individuals with <0.1% African within the 70–79-age cohort (Table 2).

**Table 3:** Test of increase in the odds of any abnormality with increasing percent of African Ancestry (AA)

	Odds Ratio (95% CI) associated with 10% increase in percent of African Ancestry	FDR adjusted *p* value
Trisomy 3	0.98 (0.94, 1.03)	0.542
Trisomy 7	0.97 (0.92, 1.01)	0.272
Trisomy 9	0.99 (0.95, 1.03)	0.542
Trisomy 11	0.96 (0.92, 1)	0.272
Trisomy 13	0.13 (0, 33.45)	0.542
Trisomy 15	0.95 (0.91, 0.99)	0.077
Trisomy 17	1.03 (0.96, 1.1)	0.542
t(4;14)	0.98 (0.91, 1.05)	0.596
t(6;14)	0.94 (0.78, 1.12)	0.542
t(11;14)	1.03 (0.99, 1.08)	0.272
t(14;16)	1.11 (1.02, 1.2)	0.077
t(14;20)	1.10 (0.96, 1.26)	0.272
t(11;14) or t(14;16) or t(14;20)	1.06 (1.02, 1.11)	0.056
other IgH	0.94 (0.87, 1.01)	0.272
TP53 deletion / Monosomy 17	0.95 (0.88, 1.01)	0.272
13q deletion or Monosomy 13	0.97 (0.93, 1.01)	0.272
Any trisomy, no IgH	0.97 (0.93, 1.01)	0.272
MYC rearrangement	0.98 (0.89, 1.08)	0.688

Odds Ratio estimate in the table is associated with 10% increase. Test of statistical significance was based on logistic regression model, with adjustment of false discovery rate using Benjamini and Hochberg’s procedure (at 0.10 level)

**Table 4:** Cytogenetic abnormalities by ancestry

	African descent (*N* = 120)	European descent (*N* = 235)	Other (*N* = 526)	Total (*N* = 881)	*p* value
Abnormality by Ancestry					
*Abnormality*					
t(11;14), t(14;16), or t(14;20)	61 (50.8%)	77 (32.8%)	180 (34.2%)	318 (36.1%)	0.008
t(4;14)	8 (6.7%)	20 (8.5%)	44 (8.4%)	72 (8.2%)	0.862
t(6;14)	1 (0.8%)	4 (1.7%)	9 (1.7%)	14 (1.6%)	0.862
Other IgH	8 (6.7%)	24 (10.2%)	54 (10.3%)	86 (9.8%)	0.739
Trisomy no IgH	37 (30.8%)	97 (41.3%)	203 (38.6%)	337 (38.3%)	0.464
All Other	5 (4.2%)	13 (5.5%)	36 (6.8%)	54 (6.1%)	0.739

	African descent (*N* = 120)	European descent (*N* = 235)	Other (*N* = 526)	Total (*N* = 881)	*p* value
Trisomy by Ancestry					
Any Trisomy					0.066
No Trisomy	62 (51.7%)	91 (38.7%)	229 (43.5%)	382 (43.4%)	
Trisomy	58 (48.3%)	144 (61.3%)	297 (56.5%)	499 (56.6%)	

*P* values are based on the comparison of the indicated abnormality (versus otherwise) compared to individuals ≥80.0% African ancestry, individuals with <0.1% African (excluding Asian ancestry) and all others individuals (3-group test) and are adjusted to control the false discovery rate (FDR) using the method of Benjamini and Hochberg

**Table 5:** Progression markers by ancestry

Progression Marker	African descent	European descent	Other	Total	*p* value
Progression markers by Ancestry					
*1q* *duplication* (*of 377 tested*)					0.576
No	30 (75.0%)	80 (70.8%)	151 (67.4%)	261 (69.2%)	
Yes	10 (25.0%)	33 (29.2%)	73 (32.6%)	116 (30.8%)	
Total	*N* = 40	*N* = 113	*N* = 224	*N* = 377	
*17p del/-17* (*of 878 tested*)					0.087
No	111 (93.3%)	202 (86.3%)	475 (90.5%)	788 (89.7%)	
Yes	8 (6.7%)	32 (13.7%)	50 (9.5%)	90 (10.3%)	
Total	*N* = 119	*N* = 234	*N* = 525	*N* = 878	
*13q del/-13* (*of 881 tested*)					0.021
No	79 (65.8%)	144 (61.3%)	283 (53.8%)	506 (57.4%)	
Yes	41 (34.2%)	91 (38.7%)	243 (46.2%)	375 (42.6%)	
Total	*N* = 120	*N* = 235	*N* = 526	*N* = 881	
*MYC rearrangement* (*of 377 tested*)					0.245
No	32 (80.0%)	100 (88.5%)	200 (89.3%)	332 (88.1%)	
Yes	8 (20.0%)	13 (11.5%)	24 (10.7%)	45 (11.9%)	
Total	*N* = 40	*N* = 113	*N* = 224	*N* = 377	

*P* values are based on the comparison of the indicated abnormality (versus otherwise) compared to individuals ≥80.0% African ancestry, individuals with <0.1% African (excluding Asian ancestry) and all others individuals (3-group test) and are adjusted to control the false discovery rate (FDR) using the method of Benjamini and Hochberg

## Discussion

Elucidating the genetic mechanisms of racial disparities is a fundamental step to understanding the etiology and improving the detection and clinical outcomes of patients with monoclonal gammopathies. Here, we complement from past studies that relied on self-reported race and characterized the patients’ demographic and uniformly collected cytogenetic data in relation to genetically defined African ancestry.

Individuals with the highest African ancestry displayed a higher prevalence of *IgH* translocations, t(11;14), t(14;16), t(14;20), lower prevalence of 13q deletion/monosomy 13 and a trend towards a lower prevalence of trisomy (with or without *IgH* translocation) compared to individuals with the least African ancestry. The differences we observed were only revealed after analysis of individuals with the highest and lowest percentage of African ancestry as no significant differences in these variables were observed when adjusting the cutoff of African ancestry to >50%, a cutoff that captures approximately 97% of AA individuals from the self-reported cohort (data not shown). Interestingly, a similar approach that considered the genetic ancestry of samples from the CoMMpass trial database found that MM tumors from Africans and Europeans vary in their frequencies of some common somatic mutated MM genes^21^. However, these results were not considered in analyzing the cytogenetic data and the authors found no significant differences between the proportions of hyperdiploid (defined in their study as presence of at least three odd-numbered chromosomes) and nonhyperdiploid karyotypes among the two groups. Approximately 50% of African individuals with greater than 80% African ancestry have either a t(11;14), t(14;16) or t(14;20) (Table 4) with the majority of this category (75%) represented by individuals with t(11;14) (38.3% of entire ≥80.0% African cohort) (Supplemental table 1). As expected, this higher prevalence of t(11;14) is associated with a lower proportion of cases with any trisomy (48.3% in ≥80.0% vs. 61.3% in <0.1% African ancestry; *p* value = 0.066) (Table 4). Further, individuals with ≥80% African ancestry also displayed a lower prevalence in 17p deletion or monosomy 17 (6.7% in ≥80.0% vs. 13.7% in 0.1% African ancestry) consistent with the higher prevalence of t(11;14) and better overall survival compared to EAs^9^.

The observation of a higher prevalence of translocations such as t(11;14), t(14;16) or t(14;20) and no enrichment in other translocations such as t(4;14) and t(6;14) suggests a possible predisposition of AAs to the development of specific chromosomal rearrangements. Many B-cell translocations are a result of aberrant B-cell mechanisms including VDJ recombination, class switch recombination and somatic hypermutation mediated by mistargeted RAG1/2 or activation induced cytidine deaminase (AID) enzymes^22^. In myeloma, most 14q32 breakpoints are localized within switch regions^23^, but whether there is a common mechanism resulting specifically in formation of t(11;14), t(14;16) or t(14;20) is unclear. If Africans display an overall increased risk of development of t(11;14), for example, one could expect increased incidence of other malignancies such as mantle cell lymphoma (MCL) also characterized by t(11;14)(q13;q32). However, epidemiological studies do not support an increased incidence of MCL among individuals of African relative to European descent^24,25^. In contrast to MM, where formation of t(11;14) is mediated by errors in class switch recombination, the t(11;14) in MCL results in errors in VDJ recombination;^26^ whether these mechanistic differences contributes to differences in the predisposition between Africans and Europeans warrants further investigation.

The further utilization of ancestry informative markers for precise characterization of biologic ancestry can help elucidate the genetic mechanisms of how race contributes to health disparities, particularly in MM where it is known that AAs have a 2–3 fold higher incidence of developing this disease^3^. Although MM is generally considered a single disease entity, MM likely represents multiple diseases characterized by distinct, mutually exclusive primary cytogenetic abnormalities with differences in disease outcome. The detection of a greater prevalence of t(11;14), t(14;16) or t(14;20) as the fraction of African ancestry increases suggests an increased incidence of specific cytogenetic subtypes in AAs rather than a global increase in all subtypes. This observation was only apparent when we separated our cohort into the most extreme populations with regard to African ancestry; individuals with ≥80.0% (n = 120) African ancestry and individuals with <0.1% African (excluding Asian ancestry) (n = 235) with the majority of patients (n = 526, 60%) not included in these extreme populations due to mixed ancestry. Although many individuals in the US are of mixed ancestry, ancestral characterization of patient cohorts is required to fully understand how the role of human genetic variation associated with ancestry impacts health disparities. Future studies will include enlarging our ≥80.0% cohort and increasing the granularity of our studies with regards to specific regions within Africa. Understanding the cause of health disparities in monoclonal gammopathies has the potential to provide previously unrecognized interventions.

## Electronic supplementary material

Supplemental table 1
