# Supplementary material for: Differences in genomic abnormalities among African individuals with monoclonal gammopathies using calculated ancestry
Source: Blood Cancer J. 2018 Oct 10;8(10):96. doi: 10.1038/s41408-018-0132-1 (PMC6180134; doi:10.1038/s41408-018-0132-1)
Supplement: Supplementary file 1 — Supplemental table 1 [file 41408_2018_132_MOESM1_ESM.docx]

**Supplemental Table 1:**

**Abnormality by Ancestry**

|  | African Descent  (80% cut-off)  (N=120) | European Descent  (N=235) | Other  (N=526) | Total  (N=881) |
| --- | --- | --- | --- | --- |
| **Abnormality** |  |  |  |  |
| t(4;14) | 8 (6.7%) | 20 (8.5%) | 44 (8.4%) | 72 (8.2%) |
| t(6;14) | 1 (0.8%) | 4 (1.7%) | 9 (1.7%) | 14 (1.6%) |
| t(11;14) | 46 (38.3%) | 63 (26.8%) | 146 (27.8%) | 255 (28.9%) |
| t(14;16) | 11 (9.2%) | 9 (3.8%) | 27 (5.1%) | 47 (5.3%) |
| t(14;20) | 4 (3.3%) | 5 (2.1%) | 7 (1.3%) | 16 (1.8%) |
| Other IgH | 8 (6.7%) | 24 (10.2%) | 54 (10.3%) | 86 (9.8%) |
| Trisomy no IgH | 37 (30.8%) | 97 (41.3%) | 203 (38.6%) | 337 (38.3%) |
| All Other | 5 (4.2%) | 13 (5.5%) | 36 (6.8%) | 54 (6.1%) |
